# Awareness of Interventional Radiology Among Medical Students at Majmaah University, Saudi Arabia

**DOI:** 10.7759/cureus.52974

**Published:** 2024-01-26

**Authors:** Meshari A Alali, Waleed F Alkhaldi, Abdulaziz Abdulhamid Alaskar, Abdullatif Mohammed Alhamad, Sultan Abdulrahman A Alhassan, Abdullah F Alsaid, Rakan Yousef Alsuwayyid, Fahad Shujaa M Almutairi, Yousuf Abdulkareem Alharbi

**Affiliations:** 1 College of Medicine, Majmaah University, Al Majma’ah, SAU; 2 College of Medicine, Majmaah University, Al Majma'ah, SAU

**Keywords:** radiology, knowledge, awareness, students, interventional radiology

## Abstract

Introduction: Interventional radiology (IR) is a medical specialty that employs imaging techniques such as X-rays, ultrasound, CT scans, and MRI to guide minimally invasive procedures for diagnosing and treating a variety of medical disorders. The purpose of this investigation was to determine the level of IR awareness among medical students at Majmaah University.

Methods: The cross-sectional investigation was carried out among the medical students at Majmaah University in Saudi Arabia. A self-administered questionnaire that had been pretested was used to gather the data. SPSS Statistics (IBM Corp. IBM SPSS Statistics for Windows. Armonk, NY: IBM Corp) was used to analyze the data. The chi-square test was used to compare qualitative data, and a p-value <0.05 was considered significant.

Results: There were 202 students who participated in this study, and among them, the majority were males, 126 (62.4%), and the majority of them were pre-clinical students, 105 (52.0%). Knowledge of routinely performed procedures by IR (only by a radiologist) was assessed; 116 (57.4%) of them responded with paracontinuous transluminal coronary angioplasty, 105 (52.0%) with central venous access, and 100 (49.5%) with lower arterial limp percutaneous transluminal angioplasty, which are routinely performed procedures by the radiologists. There was no significant difference in knowledge levels between genders.

Conclusion: Increasing awareness of IR among medical students is essential to improving patient outcomes and addressing healthcare challenges. Efforts to educate and expand access to IR services must be prioritized to ensure that medical students receive a comprehensive education and that patients receive the highest quality care possible.

## Introduction

Medical imaging modalities like X-rays, ultrasound, CT scans, and MRI are used in the field of interventional radiology (IR) to guide minimally invasive treatments for the diagnosis and treatment of a wide range of illnesses [1]. Neuroradiology, pediatric radiology, nuclear radiology, hospice and palliative care, pain management, and vascular and IR are just a few of the subspecialties that are included within diagnostic radiology [2]. The images are interpreted by interventional radiologists, who also perform a variety of interventional surgical procedures such as biopsies, drainages, angioplasties, and embolization in order to find defects and diseases [3]. Diagnosing and managing in IR includes cardiac, oncological, and neurological conditions [4]. With the help of modern intervention techniques, most parts of the body are reached by image-controlled procedures [5]. By using minimally invasive techniques, IR procedures offer patients a number of benefits over traditional surgical procedures.

The World Health Organization (WHO) published a report in 2000 titled "Efficacy and Radiation Safety in Interventional Radiology" that came to the conclusion that IR application in the treatment of diseases with cardiovascular and non-vascular origins has increased in both developed and developing nations [6]. Due to the relatively recent accreditation of IR as a subspecialty, however, modules for IR-based instruction have not yet been incorporated into the undergraduate medical curriculum. In addition, if medical students do not learn about IR, they may not refer their patients for treatment to interventional radiologists. This can contribute to delays in diagnosis and treatment, resulting in potentially poorer patient outcomes. The role of IR in the treatment of numerous disorders has expanded in recent years to encompass a number of organ systems [7,8]. However, the increased demand, complicated nature, and shortage of personnel have all come along with these broader IR symptoms [9,10].

The field is expanding rapidly, but healthcare professionals, medical students, and patients lack much knowledge about it [9]. Numerous earlier studies evaluated medical students' knowledge and awareness of IR; all of them revealed that they were less knowledgeable about this specialty [10-13]. Poor exposure to IR was noted among medical students and interns in the only study conducted in Saudi Arabia [9]. IR is a dynamic and crucial medical specialty that employs various imaging modalities to guide minimally invasive procedures for both the diagnosis and treatment of a wide range of medical conditions. However, despite its significance in modern healthcare, there remains a lack of comprehensive awareness and understanding of IR among medical students, which can have significant implications for patient care and healthcare systems. This study aimed to assess and address the level of IR awareness among medical students at Majmaah University in Saudi Arabia.

## Materials and methods

This cross-sectional study was conducted among both male and female medical students at Majmaah University, College of Medicine, in Saudi Arabia, to assess their awareness regarding intervention radiology. The research aimed to investigate awareness levels of intervention radiology among final-year medical students and utilized a pre-tested and self-administered questionnaire to collect data. The study included all male and female final-year medical students from the College of Medicine who agreed to participate voluntarily. A complete enumeration technique was used, meaning that all eligible individuals were included in the study.

The questionnaire used in this study underwent a comprehensive validation process to ensure its reliability and relevance. It was initially designed in both Arabic and English to enhance accessibility for all participants. Content validity was assessed through the scrutiny of subject matter experts in the field of IR, ensuring that the questionnaire items were relevant and appropriate for the study's objectives. Subsequently, face validity was confirmed by experts, who verified that the questionnaire appeared suitable for assessing awareness and knowledge of IR among medical students. Additionally, the questionnaire demonstrated a high level of internal consistency, as indicated by a Cronbach's alpha value of 0.8, signifying that the questions consistently measured the same construct. Pre-testing with a small sample of participants not included in the final study allowed for the identification and resolution of any ambiguities or clarity-related issues. This rigorous validation process enhances confidence in the questionnaire's reliability and suitability for the study's purpose. The questionnaire was structured to assess the awareness of final-year medical students regarding specific topics or issues related to their field of study. Ethical approval for this study was obtained from the Ethics Committee of Majmaah University (approval number: MUREC-June.19/COM-2023/23-1), ensuring that the research adhered to ethical guidelines and standards. Informed consent was obtained from each participant, indicating their willingness to participate in the study. The consent process involved providing information about the study objectives, procedures, potential risks, and the assurance of data confidentiality. Only those who provided informed consent were included in the study. Data collected from the questionnaires were analyzed using SPSS Statistics (IBM Corp. IBM SPSS Statistics for Windows. Armonk, NY: IBM Corp.). The analysis primarily involved the comparison of qualitative data using the chi-square test. A p-value <0.05 was considered statistically significant, indicating a significant association between variables or responses.

The inclusion criteria for this study were defined as follows: enrollment as a final-year medical student at Majmaah University, College of Medicine, and willingness to participate, as indicated by providing informed consent. There were no specified exclusion criteria, as the study aimed to include all eligible final-year medical students who agreed to participate. To ensure the privacy and confidentiality of the study participants, all collected data were kept confidential. The data collected were used solely for this study and were not disclosed or shared with any unauthorized individuals or organizations.

## Results

There were 202 students who participated in this study; among them, 126 (62.4%) were male and 76 (37.5%) were female. The majority of them were pre-clinical term final-year students; 105 (52.0%) and 97 (48.0%) were clinical term final-year students. About 142 (70.3%) students have completed or plan to have an elective radiology rotation. Only 52 (25.7%) students were seen as patients who were treated by an interventional radiologist. Ninety-seven (48%) of the participants knew what IR is, and 77 (38.1%) showed interest in considering a career in IR. Eighty-five (42.7%) students were considered for a career in radiology. Self-reported knowledge of IR was assessed and found that 43 (21.3%) of them had "no knowledge," 40 (19.8%) had "poor knowledge," 80 (39.6%) had "adequate knowledge," 30 (14.9%) had "good," and only nine (4.5%) had "excellent knowledge," as shown in Table 1.

**Table 1:** General characteristics and awareness of IR among participants (n=202) IR: interventional radiology

Variable	Category	Frequency (n=202)	Percent
Gender	Male	126	62.4
	Female	76	37.6
Which year are you currently in?	Pre-clinical term	105	52.0
	Clinical term	97	48.0
Completed or plan to have an elective radiology rotation?	No	142	70.3
	Yes	60	29.7
Have you seen patients who were treated by an interventional radiologist?	No	116	57.4
	Yes	52	25.7
	Not sure	34	16.8
Do you know what IR specialty is?	No	105	52.0
	Yes	97	48.0
Have you ever seen or heard of what IR doctors do?	No	91	45.0
	Yes	111	55.0
Do you know much about radiology?	No	114	56.4
	Yes	88	43.6
Would you consider a career in radiology?	No	117	57.9
	Yes	85	42.1
Would you consider a career in IR?	No	125	61.9
	Yes	77	38.1
Have you had any teaching/lectures/cases about IR?	No	125	61.9
	Yes	77	38.1
Self-reported knowledge of IR as compared with subjects?	No knowledge	43	21.3
	Poor	40	19.8
	Adequate	80	39.6
	Good	30	14.9
	Excellent	9	4.5

Knowledge of routinely performed procedures by interventional radiologists was assessed. The most prevalent procedure, with 116 (57.4%) respondents, was paracontinuous transluminal coronary angioplasty, followed by central venous access with 105 (52.0%) respondents, and lower arterial limb percutaneous transluminal angioplasty with 100 (49.5%) respondents. These were identified as routinely performed procedures by the radiologists (Table 2).

**Table 2:** Do you know which of these procedures are routinely performed by IR (only by a radiologist)? IR: interventional radiology

Variable	Frequency	Percent
1. Paracontinuous transluminal coronary angioplasty	116	57.4
2. Aortobifemoral bypass	71	35.1
3. Hemodialysis arteriovenous fistula	80	39.6
4. Central venous access	105	52.0
5. Lower arterial limp percutaneous transluminal angioplasty	100	49.5

Knowledge of routinely performed procedures in IR was also assessed. It was observed that the most common procedures routinely performed by IR included endovascular aneurysm repair (EVAR) for the treatment of abdominal aortic aneurysm, with 110 (54.5%) respondents, and image-guided core biopsy, also with 110 (54.5%) respondents. This was followed by tumoral radiofrequency ablation with 102 (50.5%) respondents and vertebroplasty with 95 (47.0%) respondents, as shown in Table 3.

**Table 3:** Do you know which of these procedures are usually done by IR IR: interventional radiology

Variable	Frequency	Percent
1. Paracontinuous transluminal coronary angioplasty	116	57.4
2. Aortobifemoral bypass	71	35.1
3. Hemodialysis arteriovenous fistula	80	39.6
4. Central venous access	105	52.0
5. Lower arterial limp percutaneous transluminal angioplasty	100	49.5

The association of knowledge levels between genders was observed to be not significant regarding completion or planning to have an elective radiology rotation, with a chi-square value of 2.114 and a p=0.146. Similarly, the association was not significant for knowing the IR specialty (chi-square = 0.189, p=0.664), knowing much about radiology (chi-square = 0.068, p=0.794), or having any teaching, lectures, or cases about IR (chi-square = 1.452, p=0.228). The only significant association observed in the knowledge level between genders considering a career in IR was p=0.037, as shown in Table 4.

**Table 4:** Association of knowledge levels between genders *: statistically significant, IR: interventional radiology

Variable		Gender		Total	chi-square, p-value
		Male	Female		
Completed or plan to have an elective radiology rotation?	No	84 (66.7%)	58 (76.3%)	142 (70.3%)	2.114, 0.146
	Yes	42 (33.3%)	18 (23.7%)	60 (29.7%)	
Have you seen patients who were treated by an IR?	No	65 (51.6%)	51 (67.1%)	116 (57.4%)	4.672, 0.097
	Yes	37 (29.4%)	15 (19.7%)	52 (25.7%)	
	Not sure	24 (19.0%)	10 (13.2%)	34 (16.8%)	
Do you know what IR specialty is?	No	64 (50.8%)	41 (53.9%)	105 (52.0%)	0.189, 0.664
	Yes	62 (49.2%)	35 (46.1%)	97 (48.0%)	
Have you ever seen or heard of what IR doctors do?	No	59 (46.8%)	32 (42.1%)	91 (45.0%)	0.427, 0.514
	Yes	67 (53.2%)	44 (57.9%)	111 (55.0%)	
Do you know much about radiology?	No	72 (57.1%)	42 (55.3%)	114 (56.4%)	0.068, 0.794
	Yes	54 (42.9%)	34 (44.7%)	88 (43.6%)	
Would you consider a career in radiology?	No	74 (58.7%)	43 (56.6%)	117 (57.9%)	0.090, 0.764
	Yes	52 (41.3%)	33 (43.4%)	85 (42.1%)	
Would you consider a career in IR?	No	71 (56.3%)	54 (71.1%)	125 (61.9%)	4.345, 0.037*
	Yes	55 (43.7%)	22 (28.9%)	77 (38.1%)	
Have you had any teaching/lectures/cases about IR?	No	82 (65.1%)	43 (56.6%)	125 (61.9%)	1.452, 0.228
	Yes	44 (34.9%)	33 (43.4%)	77 (38.1%)	
Self-reported knowledge of IR as compared with subjects?	No knowledge	23 (18.3%)	20 (26.3%)	43 (21.3%)	6.054, 0.195
	Poor	26 (20.6%)	14 (18.4%)	40 (19.8%)	
	Adequate	47 (37.3%)	33 (43.4%)	80 (39.6%)	
	Good	24 (19.0%)	6 (7.9%)	30 (14.9%)	
	Excellent	6 (4.8%)	3 (3.9%)	9 (4.5%)	

There were no significant differences in the response observed in the knowledge levels routinely performed by IR. The p-value recorded in paracontinuous transluminal coronary angioplasty was 0.116, aortobifemoral bypass was 0.192, hemodialysis arteriovenous fistula was 0.080, central venous access was 0.110, and lower arterial limp percutaneous transluminal angioplasty was 0.204, as shown in Table 5.

**Table 5:** Association of knowledge level on procedure routinely performed by IR (only by a radiologist) between genders *: statistically significant, IR: interventional radiology

Variable		Gender		Total	chi-square, p-value
		Male	Female		
1. Paracontinuous transluminal coronary angioplasty	No	59 (46.8%)	27 (35.5%)	86 (42.6%)	2.476, 0.116
	Yes	67 (53.2%)	49 (64.5%)	116 (57.4%)	
2. Aortobifemoral bypass	No	86 (68.3%)	45 (59.2%)	131 (64.9%)	1.701, 0.192
	Yes	40 (31.7%)	31 (40.8%)	71 (35.1%)	
3. Hemodialysis arteriovenous fistula	No	82 (65.1%)	40 (52.6%)	122 (60.4%)	3.071, 0.080
	Yes	44 (34.9%)	36 (47.4%)	80 (39.6%)	
4. Central venous access	No	66 (52.4%)	31 (40.8%)	97 (48.0%)	2.552, 0.110
	Yes	60 (47.6%)	45 (59.2%)	105 (52.0%)	
5. Lower arterial limp percutaneous transluminal angioplasty	No	68 (54.0%)	34 (44.7%)	102 (50.5%)	1.616, 0.204
	Yes	58 (46.0%)	42 (55.3%)	100 (49.5%)	

Significant differences in knowledge levels were observed regarding routinely performed procedures. A large number of female participants responded, with 47 (61.8%) stating tumoral radiofrequency ablation, 49 (64.5%) indicating EVAR treatment of abdominal aortic aneurysm, and 50 (65.8%) mentioning image-guided core biopsy as the routinely performed procedures, all with p<0.05, as shown in Table 6.

**Table 6:** Association of knowledge level on procedure routinely performed by IR between genders *: statistically significant, IR: interventional radiology

Variable		Gender		Total	chi-square, p-value
		Male	Female		
1. Vertebroplasty	No	66 (52.4%)	41 (53.9%)	107 (53.0%)	0.047, 0.829
	Yes	60 (47.6%)	35 (46.1%)	95 (47.0%)	
2. Tumoural radiofrequency ablation	No	71 (56.3%)	29 (38.2%)	100 (49.5%)	6.276, 0.012*
	Yes	55 (43.7%)	47 (61.8%)	102 (50.5%)	
3. EVAR treatment of abdominal aortic aneurysm	No	65 (51.6%)	27 (35.5%)	92 (45.5%)	4.931, 0.026
	Yes	61 (48.4%)	49 (64.5%)	110 (54.5%)	
4. Percutaneous nephrostomy	No	83 (65.9%)	42 (55.3%)	125 (61.9%)	2.262, 0.133
	Yes	43 (34.1%)	34 (44.7%)	77 (38.1%)	
5. Image-guided core biopsy	No	66 (52.4%)	26 (34.2%)	92 (45.5%)	6.311, 0.012*
	Yes	60 (47.6%)	50 (65.8%)	110 (54.5%)	

The comparison of knowledge levels between pre-clinical and clinical-year students was also assessed. A significantly greater number of females, 34 (35.1%), responded that they have seen patients treated by IR compared to males, 18 (17.1%) (p<0.001). Additionally, a significantly higher number of females, 60 (61.9%), knew about IR, with 68 (70.1%) of them having seen or heard of what IR doctors do, and 52 (53.6%) of the females knew about radiology (p<0.05), as shown in Table 7.

**Table 7:** Association of knowledge levels between participants of different study years *: statistically significant, IR: interventional radiology

Variables		Pre-clinical year	Clinical year	Total	chi-square, p-value
Completed or plan to have an elective radiology rotation?	No	69 (65.7%)	73 (75.3%)	142 (70.3%)	2.199, 0.138
	Yes	36 (34.3%)	24 (24.7%)	60 (29.7%)	
Have you seen patients who were treated by an interventional radiologist?	No	60 (57.1%)	56 (57.7%)	116 (57.4%)	16.535, <0.001*
	Yes	18 (17.1%)	34 (35.1%)	52 (25.7%)	
	Not sure	27 (25.7%)	7 (7.2%)	34 (16.8%)	
Do you know what IR specialty is?	No	68 (64.8%)	37 (38.1%)	105 (52.0%)	14.312, <0.001*
	Yes	37 (35.2%)	60 (61.9%)	97 (48.0%)	
Have you ever seen or heard of what IR doctors do?	No	62 (59.0%)	29 (29.9%)	91 (45.0%)	17.308, <0.001*
	Yes	43 (41.0%)	68 (70.1%)	111 (55.0%)	
Do you know much about radiology?	No	69 (65.7%)	45 (46.4%)	114 (56.4%)	7.657, 0.006*
	Yes	36 (34.3%)	52 (53.6%)	88 (43.6%)	
Would you consider a career in radiology?	No	58 (55.2%)	59 (60.8%)	117 (57.9%)	0.646, 0.422
	Yes	47 (44.8%)	38 (39.2%)	85 (42.1%)	
Would you consider a career in IR?	No	61 (58.1%)	64 (66.0%)	125 (61.9%)	1.329, 0.249
	Yes	44 (41.9%)	33 (34.0%)	77 (38.1%)	
Have you had any teaching/lectures/cases about IR?	No	71 (67.6%)	54 (55.7%)	125 (61.9%)	3.052, 0.081
	Yes	34 (32.4%)	43 (44.3%)	77 (38.1%)	
Self-reported knowledge of IR as compared with subjects?	No knowledge	28 (26.7%)	15 (15.5%)	43 (21.3%)	4.315, 0.365
	Poor	18 (17.1%)	22 (22.7%)	40 (19.8%)	
	Adequate	41 (39.0%)	39 (40.2%)	80 (39.6%)	
	Good	14 (13.3%)	16 (16.5%)	30 (14.9%)	
	Excellent	4 (3.8%)	5 (5.2%)	9 (4.5%)	

The p-values observed in the knowledge levels of procedures routinely performed by IR were as follows: paracontinuous transluminal coronary angioplasty (0.038), aortobifemoral bypass (0.148), hemodialysis arteriovenous fistula (0.108), and central venous access (0.656) between the students of pre-clinical years and clinical years. No significant difference was observed in the knowledge levels of procedures routinely performed by IR between the students of pre-clinical and clinical years, with p>0.05, as shown in Table 8.

**Table 8:** Association of knowledge levels on procedures routinely performed by IR (only by a radiologist) between study years *: statistically significant, IR: interventional radiology

Variable		Pre-clinical year	Clinical year	Total	chi-square, p-value
1. Paracontinuous transluminal coronary angioplasty	No	52 (49.5%)	34 (35.1%)	86 (42.6%)	4.319, 0.038*
	Yes	53 (50.5%)	63 (64.9%)	116 (57.4%)	
2. Aortobifemoral bypass	No	73 (69.5%)	58 (59.8%)	131 (64.9%)	2.094, 0.148
	Yes	32 (30.5%)	39 (40.2%)	71 (35.1%)	
3. Hemodialysis arteriovenous fistula	No	69 (65.7%)	53 (54.6%)	122 (60.4%)	2.586, 0.108
	Yes	36 (34.3%)	44 (45.4%)	80 (39.6%)	
4. Central venous access	No	52 (49.5%)	45 (46.4%)	97 (48.0%)	0.198, 0.656
	Yes	53 (50.5%)	52 (53.6%)	105 (52.0%)	
5. Lower arterial limp percutaneous transluminal angioplasty	No	53 (50.5%)	49 (50.5%)	102 (50.5%)	0.001, 0.996
	Yes	52 (49.5%)	48 (49.5%)	100 (49.5%)	

There was no significant association of knowledge levels on procedures routinely performed by IR. The p-value of vertebroplasty was 0.340, tumoral radiofrequency ablation was 0.577, and image-guided core biopsy was 0.143. The significance was that EVAR treatment of abdominal aortic aneurysm was 0.001 and percutaneous nephrostomy was 0.020 between study years, as shown in Table 9.

**Table 9:** Association of knowledge levels on procedure routinely performed by IR between study years *: statistically significant, EVAR: endovascular aneurysm repair, IR: interventional radiology

Variables		Pre-clinical year	Clinical year	Total	chi-square, p-value
1. Vertebroplasty	No	59 (56.2%)	48 (49.5%)	107 (53.0%)	0.910, 0.340
	Yes	46 (43.8%)	49 (50.5%)	95 (47.0%)	
2. Tumoral radiofrequency ablation	No	50 (47.6%)	50 (51.5%)	100 (49.5%)	0.311, 0.577
	Yes	55 (52.4%)	47 (48.5%)	102 (50.5%)	
3. EVAR treatment of abdominal aortic aneurysm	No	60 (57.1%)	32 (33.0%)	92 (45.5%)	11.860, 0.001*
	Yes	45 (42.9%)	65 (67.0%)	110 (54.5%)	
4. Percutaneous nephrostomy	No	73 (69.5%)	52 (53.6%)	125 (61.9%)	5.414, 0.020*
	Yes	32 (30.5%)	45 (46.4%)	77 (38.1%)	
5. Image-guided core biopsy	No	53 (50.5%)	39 (40.2%)	92 (45.5%)	2.144, 0.143
	Yes	52 (49.5%)	58 (59.8%)	110 (54.5%)	

## Discussion

IR is an evolving medical specialty with diverse applications in healthcare, including oncology, cardiovascular medicine, trauma, and urology [14,15]. However, IR faces challenges such as a lack of awareness and a shortage of trained professionals [16]. The future generation of interventional radiologists and referring physicians, medical residents and students play a pivotal role in shaping the field's growth [17]. Thus, this study, conducted at Majmaah University, aimed to assess the level of knowledge regarding IR among medical students.

In Saudi medical schools, radiology curricula vary, with some universities offering it as a standalone course and others integrating it into various medical subjects [11]. Radiology education, particularly in pre-clinical courses, has gained significance in recent years [18,19]. Expanding students' awareness of this profession could substantially increase their interest in radiology and IR. However, few studies have examined the difference in IR knowledge between pre-clinical and clinical years [20].

The study revealed that among the participants, 62.4% were male and 37.6% were female. Further analysis showed that 70.3% of students had completed or planned to undertake an elective radiology rotation, while 42.7% had considered a career in radiology. Self-reported knowledge of IR indicated that 21.3% had "no knowledge," 19.8% had "poor knowledge," 39.6% had "adequate knowledge," 14.9% had "good knowledge," and only 4.5% had "excellent knowledge," highlighting a poor awareness of this specialty. No statistically significant difference was observed in knowledge levels regarding routine IR procedures, including paracontinuous transluminal coronary angioplasty, aortobifemoral bypass, hemodialysis arteriovenous fistula, and central venous access, between pre-clinical and clinical year students.

Similar findings were observed in a study conducted in Saudi Arabia, where 52% of students exhibited poor knowledge of IR [11]. These local results align with research from Ireland [14], where 62% of medical students had limited knowledge of IR, as well as studies from England [21] and Canada [12], reporting minimal awareness of IR as a specialty among 55.5% and 52% of students, respectively. In a cross-sectional study at King Abdulaziz University, Jeddah, Saudi Arabia, involving 542 medical students, 36.7% expressed low confidence in their understanding of IR, while 15.7% reported a complete lack of knowledge in this field. Only 16.1% considered a career in radiology, with a significant reason for not considering IR being a lack of knowledge (42.9%). Clerkship students exhibited more interest in and exposure to IR than pre-clerkship students (73.0% vs. 55.7%) [2].

Another cross-sectional study conducted in Saudi Arabia, comprising 119 medical interns and students from King Khalid University in Abha, revealed that only 40% had fulfilled or intended to complete a radiology elective rotation. Additionally, 38% of respondents expressed openness to pursuing a career in IR, with a lack of information cited as the primary barrier to career consideration (43%). Notably, only 33% correctly identified the training path for interventional radiologists, while 81% and 74% incorrectly believed that interventional radiologists performed femoral-popliteal bypass and heart angioplasty, respectively [11].

IR holds immense potential for improving patient outcomes and addressing significant healthcare challenges. However, many medical students remain unfamiliar with this field and its minimally invasive procedures, which offer reduced pain and complications compared to traditional surgery. Enhancing awareness of IR among medical students is vital to improving patient care quality and reducing healthcare costs.

Limitations of this study include its single-center design, potentially limiting generalizability, reliance on self-reported knowledge assessments susceptible to response bias, a cross-sectional approach without capturing knowledge changes over time, a small sample size impacting result precision, and a lack of qualitative data to delve into students' knowledge levels and perceptions of IR. Furthermore, the study did not explore specific curriculum details or potential interventions for enhancing awareness among medical students.

## Conclusions

This study sheds light on the awareness and knowledge levels of IR among medical students at Majmaah University, College of Medicine. The findings revealed a significant gap in IR awareness and knowledge, with a majority of students reporting "no knowledge" or "poor knowledge" of this crucial medical specialty. Additionally, significant disparities in knowledge levels between pre-clinical and clinical year students were observed, with clinical year students exhibiting higher awareness. This study underscores the importance of integrating comprehensive IR education into the medical curriculum to bridge the awareness gap and nurture interest among future healthcare professionals. Further efforts are needed to enhance IR awareness and promote its role in modern healthcare.

## Figures and Tables

**Figure 1:**
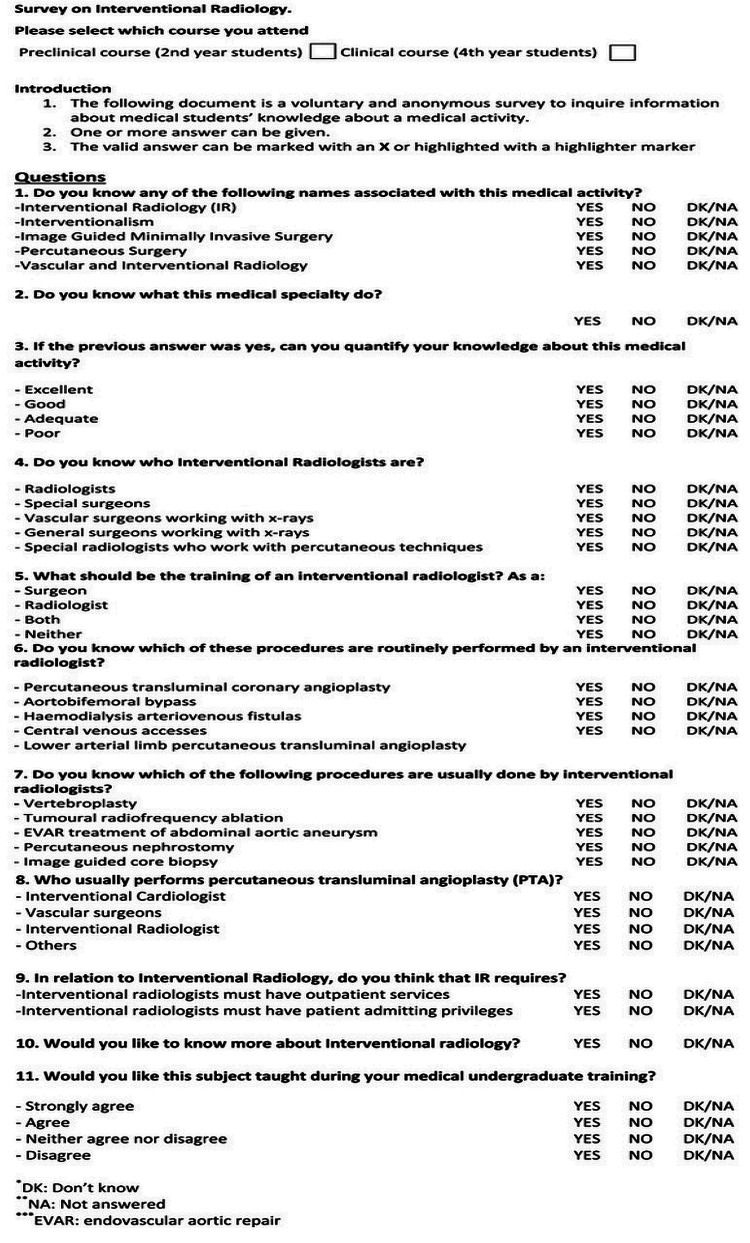
Survey questionnaire
